# Detection of network motifs using three-way ANOVA

**DOI:** 10.1371/journal.pone.0201382

**Published:** 2018-08-06

**Authors:** Pegah Tavakkolkhah, Ralf Zimmer, Robert Küffner

**Affiliations:** 1 Department of Informatics, Ludwig-Maximilians-Universität München, München, Germany; 2 Icahn School of Medicine at Mount Sinai, New York, NY, United States of America; King’s College London, UNITED KINGDOM

## Abstract

**Motivation:**

Gene regulatory networks (GRN) can be determined via various experimental techniques, and also by computational methods, which infer networks from gene expression data. However, these techniques treat interactions separately such that interdependencies of interactions forming meaningful subnetworks are typically not considered.

**Methods:**

For the investigation of network properties and for the classification of different (sub-)networks based on gene expression data, we consider biological network motifs consisting of three genes and up to three interactions, e.g. the cascade chain (CSC), feed-forward loop (FFL), and dense-overlapping regulon (DOR). We examine several conventional methods for the inference of network motifs, which typically consider each interaction individually. In addition, we propose a new method based on three-way ANOVA (ANalysis Of VAriance) (3WA) that analyzes entire subnetworks at once. To demonstrate the advantages of such a more holistic perspective, we compare the ability of 3WA and other methods to detect and categorize network motifs on large real and artificial datasets.

**Results:**

We find that conventional methods perform much better on artificial data (AUC up to 80%), than on real E. coli expression datasets (AUC 50% corresponding to random guessing). To explain this observation, we examine several important properties that differ between datasets and analyze predicted motifs in detail. We find that in case of real networks our new 3WA method outperforms (AUC 70% in E. coli) previous methods by exploiting the interdependencies in the full motif structure. Because of important differences between current artificial datasets and real measurements, the construction and testing of motif detection methods should focus on real data.

## Introduction

Inference of gene regulatory networks (GRNs) aims to improve our understanding of the cellular responses to local as well as environmental signals. The expression of a target gene (TG) can be activated or repressed via binding of proteins known as transcription factors (TF) to its promoter region. In this study, GRNs refer to directed graphs in which edges represent the regulatory effects of a TF on its TG. Several techniques [1–3] have focused on the inference of GRNs using transcriptome data. Transcriptome data is one of the main sources to detect regulatory interactions, not only because modeling the control of transcription is the main purpose of GRNs, but also due to the fact that measurements of mRNA levels are more readily available than other high throughput experiments, e.g. proteomic profiles [4].

Approaches based on measuring pairwise dependencies between TF:TG pairs assume that an interaction is more likely if the expression of a TG is correlated to the expression of the TF. Pearson’s correlation (PC; [5]), mutual information (MI; [6]) and two-way ANOVA (analysis of variance; [7]) have been successfully applied to infer pair-wise interactions. Such methods cannot distinguish between indirect and direct interactions, as relations defined by correlation are transitive. The indirect ‘interaction’ between *A* and *C* via *A* → *B* and *B* → *C* can lead to a substantial correlation between *A* and *C* and may thus imply a direct ‘interaction’ *A* → *C*, known as cascade error [8].

This is just one example indicating that testing individual interactions may not be sufficient to examine complex subnetwork behavior arising from direct and indirect regulatory effects. Here, we argue that complete subnetworks have to be considered. As a first step in that direction, we focus on the simplest form of such subnetworks composed of direct and indirect interactions between two TFs and a TG. Such subnetworks or building blocks appear significantly more frequent in networks compared to random networks and are called network motifs or motifs for short [9, 10] (see below). We propose a novel method based on three-way ANOVA (3WA) to examine and distinguish such motifs. We demonstrate its advantages by comparatively evaluating 3WA and previously published methods on both artificial and real datasets.

### Network motifs

**Fig 1:**
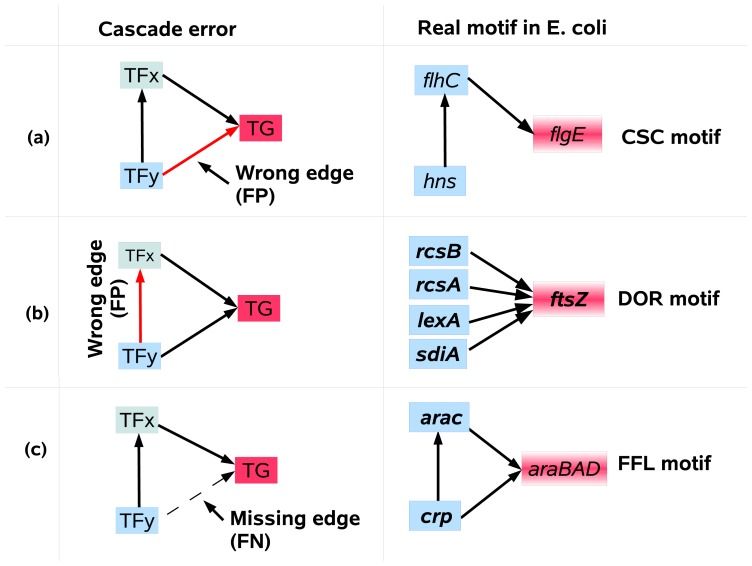
Three common cases of cascade error. Three common cases of cascade error, i.e misinterpretation of direct effects for indirect and vice versa, in the inference of CSC, DOR, and FFL motifs. The red arrow represents a false positive (FP) edge, i.e an edge is inferred when it does not exist in the gold standard, and the dotted arrow shows a false negative (FN) edge, i.e a true edge has not been inferred. The right side shows for each subnetwork a corresponding example in the gene regulatory network of E. coli.

Feed-forward loop (FFL), cascade chain (CSC), and dense-overlapping regulon (DOR) are examples of network motifs (Fig 1). A CSC motif (Fig 1a) (also known as regulator chain; [11]) refers to a chain of two or more regulators, in which one regulator binds to the promoter of the second regulator, and, the second binds to the promoter of a third regulator, and so on. Simon et al. [12] showed that in the yeast cell cycle the transcriptional activators functioning at one phase of the cell cycle regulate the activators required for entry into the next phase of the cell cycle. The DOR motif (Fig 1b), also known as multi-input motif, is a layer of overlapping interactions between operons and a group of input TFs. For instance, the *fts* operon, which plays essential roles in the regulation of cell division, is regulated by several TFs and forms a DOR motif [13]. FFL motifs (Fig 1c) are shown to be able to filter short input signals, and also allow a rapid system shutdown [14].

Several studies [14–16] have analyzed whether there is a relationship between motif structure and its function, for instance based on the coherence of motifs. In contrast to incoherent FFL motifs, coherent FFL motifs have the same effect sign (either repression or activation) on both the direct and indirect regulation branches. Dunlop et al. [17] showed that incoherent FFL motifs reduce circuit-extrinsic noise, while coherent FFLs amplify such noise.

The behavior and topology of a motif is often defined based on the mRNA expression pattern of the TG and TF-coding genes. This requires a careful consideration of the regulatory mechanisms involved. For example, competition, synergy or the cooperation of multiple regulators might lead to complex expression patterns. Also, post-translational regulation of TFs may modify the binding of a TF to its targets. Hence, not all regulatory effects will be immediately interpretable on the transcriptional level. In addition, other factors such as environmental signals as well as regulators outside the motif context [18] affect the expression of TGs and TF-coding genes and lead to more complex behaviors. Ingram et al. [19] studied the dynamics of bi-fan motifs in which two TFs jointly regulate two TGs, and showed that the same motif may exhibit different behaviors depending on the experimental condition.

The behavior of a motif is also dependent on the strength of its pairwise interactions, e.g. an FFL motif with a weak TF:TF interaction might behave like a DOR motif. In a weak interaction, the activity of the regulator has only little effect on the expression of the target. Huang et al. [9] showed that both in *E*. *coli* and *S*. *cerevisiae*, in most of the motifs one or more of the interactions are weak. In addition, several studies confirm that the effect sign of interactions are important for the behavior of FFL motifs [17, 20–22].

### Related work

In this paper, we propose to detect, categorize and examine three basic subnetworks, CSC, FFL, and DOR using expression data. The ability to distinguish these motifs can for instance serve to reduce the rate of cascade error, which refers to misinterpretation of direct effects for indirect effects and vice versa. Cascade error is illustrated in Fig 1.

Several approaches propose to at least partially consider subnetworks with three or more genes to distinguish direct from indirect effects. A straightforward method is measuring conditional correlation, which is the residual pairwise correlation after conditioning over one or several other genes. de la Fuente et al. [23] used first and second order conditional Pearson’s correlation and first order conditional Spearman’s correlation to distinguish direct from indirect interactions. Watkinson et al. [24] and Luo et al. [25] used three-way mutual information to measure the joint effect of two TFs on the expression of a TG in addition to their individual effects. ARACNe [26] and PCA-CMI [27] both begin with a network in which all edges have a mutual information (MI) higher than a specific threshold. ARACNe removes the edge with the lowest dependency in all fully connected three-gene structures. In all fully connected 3-gene structures (e.g. FFLs), the edge with the lowest dependency is removed if its dependency is substantially lower than that of the two other edges. PCA-CMI, on the other hand, computes higher order conditional mutual information, i.e it is not limited to the joint effect of only two TFs, to remove edges between pairs that are not highly dependent when considering one or several other TFs. In all procedures described above, edges are removed that may or may not be false positives resulting from indirect interactions. However, it is often unclear whether these methods are actually able to distinguish CSC and FFL motifs or whether the adaptations only serve to change a method’s relative preference by unspecifically trading CSC for FFL motifs or vice versa [28].

## Results

The present study focuses on the detection of network motifs consisting of three genes and up to three interactions. The analysis is performed based on several real datasets and an artificial dataset.

We propose a novel approach for this task and compare it against published methods. Before discussing the results on the network motifs we analyze the properties of individual interactions that are relevant for the more complicated task of motif detection. We used known pairwise as well as conditional dependence metrics. Conditional dependencies are primarily applied to help distinguishing between direct and indirect interactions.

### Differences between real and the artificial datasets

#### Enrichment of correlation of interacting gene pairs compared to arbitrary pairs

The performance of inference methods is often dependent on how well interacting pairs can be distinguished from random pairs based on measuring the dependency between their expression profiles. PC for instance is frequently used as a measure of dependency within inference approaches. Therefore, we examine networks in terms of random as well as interacting pairs (TF:TF or TF:TG) to derive normalized dependency distributions (as binned histograms), where TG refers to genes that are always targets while TFs can be transcription factors as well as target genes.

**Fig 2:**
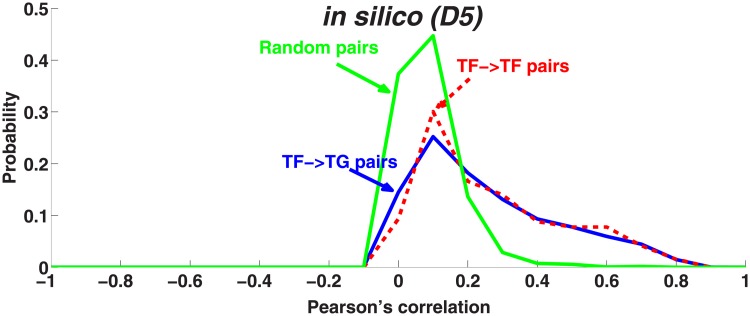
Correlation of random pairs vs. interacting pairs. Correlation of random pairs vs. interacting pairs. The distribution of correlation for random pairs (green-solid line) and interacting pairs (TF:TG pairs (blue-solid line), TF:TF pairs (red-dashed line)) in the artificial dataset were normalized to unit area. The x-axis and y-axis show PC and probability, respectively.

**Fig 3:**
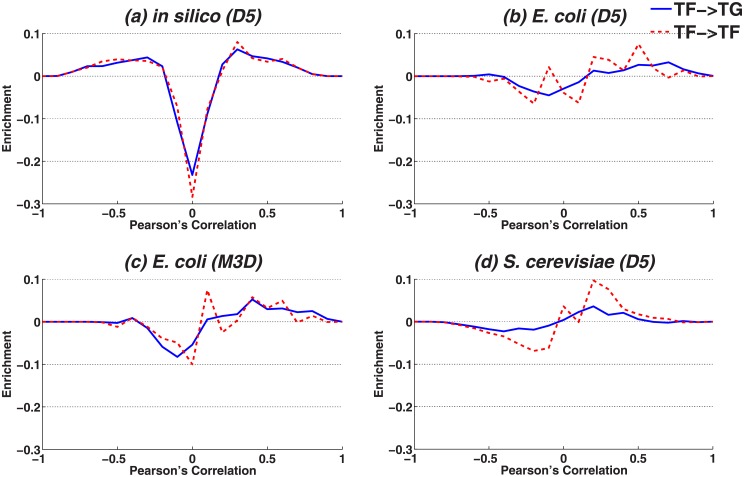
Difference histograms for correlations. Difference histograms for correlations. We display the difference between two histograms (*i*, e.g. red or blue line, Fig 2) and (*ii*, e.g. green line, Fig 2) by subtracting (*i*)–(*ii*). As the probability of interacting versus non-interacting pairs with a given correlation might be decreased or increased, difference histograms display positive or negative values, respectively. The plots compare the difference distribution of TF:TG (solid) and TF:TF (dashed) in the real (b)-(d) and the artificial datasets (a).

**Fig 4:**
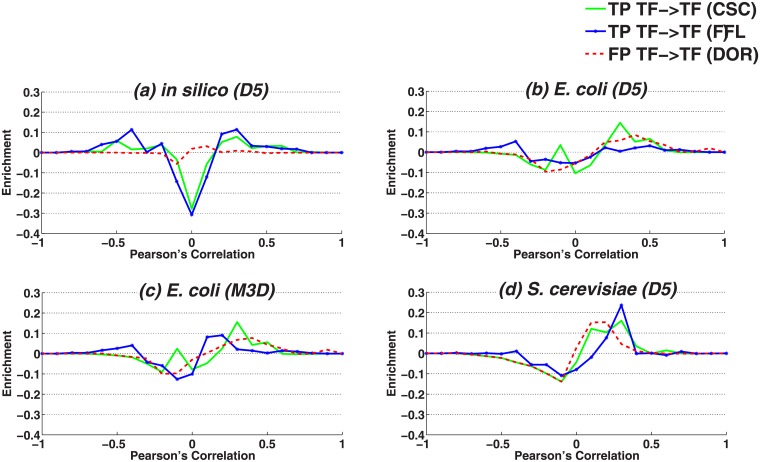
Correlation vs. causal relationships. Shown here are individual difference histograms (compare Fig 3) for DOR, CSC and FFL motifs in real and the artificial datasets.

These histograms are shown in Fig 2 for the artificial dataset. We consider two distributions, (i) of true interactions (TF:TF, TF:TG), and (ii) of random pairs. Distributions of random interactions peak at a correlation value of 0.0, which implies that random pairs typically exhibit no correlation. We then plot the bin-wise difference of these two distributions (*i*–*ii*) for each network in Figs 3 and 4. Positive or negative values of this difference can now be interpreted as the enrichment or the depletion, respectively, for observing true interactions at given PC intervals.

As depicted in Fig 3a, the artificial dataset shows enrichment for correlation values above 0.2 or below -0.2, and depletion for correlation values around zero. Hence, it is very likely that interacting gene pairs show higher absolute correlation compared to arbitrary gene pairs, making it easy to distinguish them. Consequently, motifs should be distinguishable by comparing their individual characteristic edges, e.g. DOR motifs can be distinguished from FFL motifs by comparing their TF:TF pairs, ignoring other interactions involved. It is also very interesting that in contrast to the real datasets, the distributions for TF:TF and TF:TG pairs are hardly distinguishable in the artificial data.

By comparing the artificial dataset with real datasets (Fig 3a vs. Fig 3b and 3c), we observe two main differences. First, the range of enrichment/depletion (ordinate) in the artificial dataset is much more pronounced in comparison to real datasets, i.e in case of real datasets dependencies between interacting pairs cannot as easily be distinguished from random pairs. In case of *S*. *cerevisiae*, it is even more obvious as the pairs with the highest correlation do not exhibit an increased chance to represent true interactions. Second, in the real datasets the histograms for TF:TF pairs and TF:TG pairs show substantially different properties, which cannot be observed in the artificial dataset. This could indicate that these two types of interactions should be treated differently.

Next, we analyzed interacting TF:TF pairs in CSC and FFL motifs as well as non-interacting TF:TF pairs in DOR motifs, to examine correlation distributions in the context of motifs (Fig 4). In the artificial dataset, the interacting TF:TF pairs in CSC and FFL are enriched (correlation values above 0.2 or below -0.2) while the non-interacting TF:TF pairs in DOR are not. This indicates that DOR motifs can be distinguished from CSC and FFL only by comparing their characteristic edge (Fig 4a). In contrast to the artificial dataset, real datasets exhibit much less enrichment (Fig 4b and 4c), which is very similar for DORs, across the three interactions.

In contrast to the artificial dataset, we expect that an analysis of individual edges may be insufficient to distinguish motifs in the real datasets as interacting and non-interacting pairs are more difficult to separate.

#### The sign of interactions

As mentioned in the introduction, the sign of the interactions (activation/repression) is considered important for understanding the behavior of motifs. In the following, we analyzed *E*. *coli* expression profiles with respect to the type of regulatory interactions (activation/repression) that we extracted from the annotations within RegulonDB.

**Fig 5:**
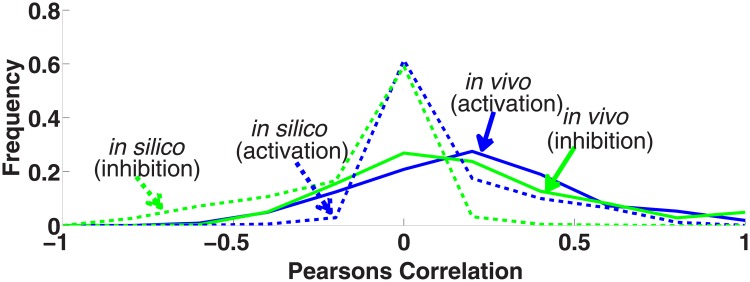
The distribution of Pearson’s correlation based on the sign of interactions. The distribution of PC based on the sign of interactions (activating or repressing) for the artificial (solid) and real (*E*. *coli*) (dashed) datasets is shown.

Theoretically, a negative PC should be an indication of a repressing interaction. As depicted in Fig 5, in the artificial data positive and negative correlations exhibit their corresponding signs in the PC distribution and their strength (positive/negative shift of PC is similar). In the *E*. *coli* dataset, the distribution of PC generally is shifted to positive values so that strong negative correlations and, thus, repressing interactions are rarely observed in our expression data.

**Fig 6:**
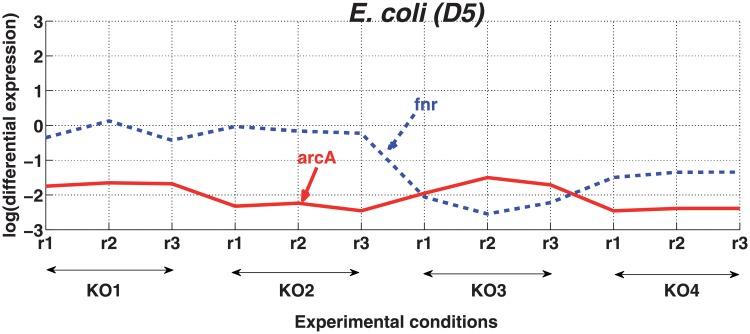
The effect of knockout of *arcA* on its target *fnr*. Four different knockout experiments of *arcA* with three replicates each (abscissa), and the response of its target gene, *fnr*, to these knockouts is depicted in the form of expression log fold-change (ordinate).

We also examined the sign of interactions based on the differential expression of a TG in response to the knockout or overexpression of its regulator. We analyzed the effect of knockout of TFs with repressing effects on their TGs. The pair *arcA*:*fnr* is an example where *fnr* is repressed by its only regulator *arcA*. In Fig 6, the expression of *fnr* in four knockout experiments, each with 3 replicates, is shown. Since *arcA* represses *fnr*, upregulation of *fnr* is expected in response to the knockout of *arcA*. However, in the first two knockout experiments the expression log fold changes of *fnr* is around zero, i.e its expression is not changed compared to the wild type experiment. In the last two knockout experiments, the expression of *fnr* is lower as compared to the wild type condition. Different reasons could result in such a pattern, for example a more complex regulatory mechanism that regulates the expression of *fnr* which might not entirely depend on *arcA*.

Overall, we conclude that it is very difficult to determine the sign of interactions from expression data, and that the coherence or incoherence of motifs cannot be analyzed in this way.

### Pairwise versus three-way methods

**Fig 7:**
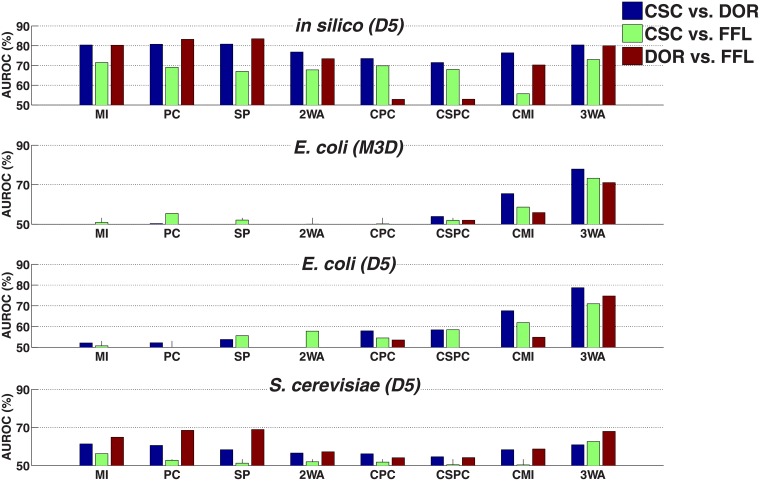
Comparison of pairwise classification results of CSC, FFL, and DOR motifs across datasets. Pairwise classification results across a range of methods (see text) in *E*. *coli* (M3D), *E*. *coli* (D5), artificial (D5), and S. cerevisiae (D5). The ordinate represents the AUROC. Since AUROC = 50% is equivalent to random guessing, values lower than 50% are not shown.

In this section, we compare the performance of 3WA and current approaches to distinguish motifs (Fig 7). Simple (i.e pairwise) methods analyze interactions individually, while 3WA and conditional methods take the entire motif into account. For each pair of motif types in turn (CSC vs. DOR, CSC vs. FFL and DOR vs. FFL), we analyze how well instances of one motif type can be recognized and distinguished from the other.

#### Artificial dataset

As shown in Fig 7, in the artificial dataset all simple approaches (MI, PC, SP, 2WA) were able to recognize all motifs with good performance. As discussed earlier in section 2.1.1, in the artificial dataset interacting and non-interacting pairs are in general easy to distinguish (Fig 3a). In particular, DOR has a characteristic edge between the two regulatory TFs, which can be used independently from other edges to distinguish the DOR from the two other motifs without considering the other edges. Fig 4a furthermore confirms that the TF:TF correlation in DOR motifs (where this interaction is absent) is indeed very low and easily distinguishable from the TF:TF interactions in both other motifs. The conditional dependency measures (conditional PC, SP and MI) showed a lower performance than their non-conditional counterparts. 3WA is performing similar to the best available approaches.

#### Real datasets

*E*. *coli* dataset. Our results on *E*. *coli* datasets were substantially different from those obtained for the artificial data. In general, performance of simple approaches as well as the conditional correlation coefficients were close to random guessing, which can be explained by the fact that the distribution of dependencies between interacting pairs overlaps largely with non-interacting pairs (Fig 3b and 3c). In addition, according to Fig 4b and 4c the distribution of pairwise dependency of TF:TF pairs in DOR motifs is similar to those of CSC and FFL motifs, i.e non-interacting TF-TF pairs in DOR cannot be distinguished from interacting TF:TF pairs in FFL and CSC simply by comparing individual pairwise dependencies.

In contrast, conditional mutual informaiton was to some extent able to distinguish between all three motifs in both *E*. *coli* datasets (D5 and M3D).

3WA performs substantially better than both simple and conditional approaches. For example, for the *E*. *coli* (D5) dataset, DOR motifs are distinguished from FFL motifs with an AUROC of 74%.

*S*. *cerevisiae* dataset. The performance of simple approaches is slightly better than random guessing (AUROC ≈ 60%) in the *S*. *cerevisiae* dataset, i.e. simple approaches perform better here than in the *E*. *coli* dataset, particularly in case of DOR vs. FFL. This might be due to the fact that in *S*. *cerevisiae*, TF:TF pairs show slightly higher correlation in both FFL and CSC motifs compared to DOR motifs (Fig 4d), which is in contrast to both *E*. *coli* datasets (Fig 4b and 4c). The lowest performance is achieved for the separation of CSC and FFL. One possible reason is that the correlation distribution of interacting TF:TG pairs overlap largely with that of non-interacting TF:TG pairs (Fig 3d). The performance of conditional methods in all classification tasks is almost near random guessing and conditional mutual informaiton is just slightly better in classifications involving DOR.

3WA has a similar performance to simple approaches, still, its performance in distinguishing CSC from FFL motifs (AUROC = 62%) is higher compared to the performance of all other approaches.

To summarize, 3WA has a higher performance in real datasets compared to standard approaches, and its performance is similar to the best other methods in the artificial dataset. Conditional approaches have a low performance in all real datasets, only conditional mutual information being slightly better. The performance of the examined existing motif classification approaches is satisfactory only in case of the artificial dataset.

### Examples of misclassifications and correct classifications

Distinguishing CSC motifs from FFL motifs is quite challenging, since only one edge differs between them. In turn, a missing interaction in the gold standard of regulatory interactions can change a FFL motif into a CSC motif, and similarly, a false positive interaction can change a CSC motif into a FFL. For example, according to interactions annotated in RegulonDB, the motif (*fadR*→*iclR*→*aceK*) is a CSC motif, i.e. no interaction has been annotated between *fadR* and *aceK*. However, based on our 3WA this motif was categorized FFL with high confidence, i.e. implying a dependency between the expression profiles of *fadR* and *aceK*. Based on Meyer et al. [29], the expression of the *ace* operon is under the transcriptional control of both *iclR* and *fadR*, providing a sufficient explanation of the observed dependency between the expression profiles of *fadR* and *aceK* and suggesting that it could indeed show the behavior of a FFL.

**Fig 8:**
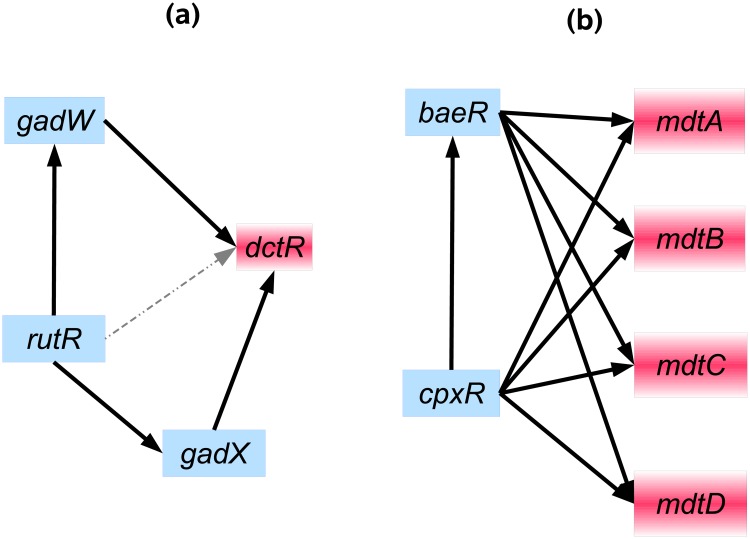
Two examples of motifs that are categorized as FFL by 3WA. Two examples of motifs that are categorized as FFL by 3WA: (a) The three genes of(*gadW*, *rutR*, *dctR*) form a CSC motif which is incorrectly categorized as FFL by 3WA. It might be due to the fact that *rutR*:*dctR* form an indirect interaction via *gadX*. True positive and false positive interactions are shown with solid and dotted lines respectively. (b) The two TFs *baeR* and *cpxR* form several FFL motifs with the *mdt* operon (*baeR*: TF, *cpxR*: TF, *mdt* operon: TG). According to annotation of RegulonDB, this group of motifs are isolated in the GRN of *E*. *coli*, i.e. 1) the two TFs are not regulated by any other TFs, and 2) the expression of *mdt* operon is only regulated by *baeR* and *cpxR*.

Another reason for misclassification of CSC and FFL motifs are indirect interaction(s) between non-interacting TF:TG pairs in CSC. For example, the CSC motif (*rutR*→*gadW*→*dctR*) (Fig 8a) has been categorized by 3WA as FFL with a high confidence score. According to RegulonDB, *rutR* forms a second cascade chain with *dctR* through *gadX*, i.e. an indirect interaction, which might result in a strong pairwise dependency between *rutR*:*dctR*. Thus, other network interactions might lead to a behavior different from what the motif would exhibit in isolation.

The FFL motifs formed by (*baeR*: TF, *cpxR*: TF, *mdt* operon) are examples of correct classification by 3WA (Fig 8b). According to RegulonDB *cpxR* does not have any regulator, and *cpxR* itself is the only regulator of *baeR*. In addition, the *mdt* operon is also only regulated by these two TFs. Because of that, these motifs seem to be rather isolated and lack a dense connection to the remaining network. As these FFL motifs are not affected by the input of other TFs, they have been recognized correctly as FFLs by 3WA.

## Discussion

Apart from individual binary interactions, network motifs consisting of just three genes and up to three interactions, are the simplest pathway building blocks. They may perform distinct functions, e.g. feed-forward loops (FFL) are considered to act as low-pass filters, removing very short signals. Motif function is thought to emerge from the exact topology, rather than from individual interactions or genes. Thus, the full structure needs to be considered for motif detection and examination. In contrast, motif detection approaches are often based on binary dependency measures such as Pearson’s correlation, and, thus, consider each interaction individually. In order to examine the interactions of three genes simultaneously, we suggested an approach based on three-way-ANOVA (3WA) to detect motifs consisting of transcription factor:target gene (TF:TG) interactions from expression data. Notably, our method can be extended to larger subnetworks of four (4WA) or five (5WA) gene motifs, of course at increased computational cost.

We compared the performance of 3WA to several published approaches, e.g. based on (conditional) Pearson’s correlation or mutual information. On the artificial data, 3WA performs similar to previous approaches. On real data, 3WA outperforms standard approaches by a large margin. 3WA also outperforms conditional approaches, which aim to discriminate direct from indirect interactions. There are several reasons why 3WA outperforms conditional methods in real data. First, TF:TG and TF:TF interactions have different properties and might show different levels of dependency. While 3WA takes this difference into account, conditional methods treat all interactions as if they are of the same type. Here, 3WA decomposes the data into 8 sum of squares, that enables a flexible modeling and, thereby, a more accurate interrogation of the motif-specific properties. Second, conditional approaches treat the replicates of the same experiment as individual measurements. In contrast, ANOVA uses the replicates to increase motif-specific signal to noise ratio. Thereby, 3WA is capable to detect weaker signals in real data in comparison to other approaches. However, it should be taken into account that ANOVA cannot be applied if replicates are not available or the data is not approximately normally distributed. Indeed, the distribution of log-fold change expression data used here is close to normal and it has been shown previously that in such cases ANOVA yields the best results among a range of methods [30].

Furthermore, the discrepancy between the performance of tools on real and simulated data can be explained partially by the fact that most standard approaches were devised, trained and tested primarily on simulated data (e.g. [23]). However, there are several important properties that differ between real and artificial datasets.

First, the detection of individual interactions is much simpler in artificial datasets as TFs and TGs show much stronger correlation for true artificial interactions as compared to other gene pairs. In the artificial data this enables the detection of motifs by simply testing each interaction individually. In contrast, in real data, correlation distributions of true and false interactions overlap substantially so that interactions are more difficult to detect. No increased correlation can be detected in TF:TF interactions that are particularly important in network motifs, so that more sensitive methods are required.

The difficulty in distinguishing true and false interaction partially explains our findings that testing single interactions is not sufficient. This might be due to the fact that TFs are subject to post-transcriptional regulation [31] in addition to transcriptional regulation. Post-transcriptional regulation is not visible on the mRNA expression level. 3WA achieves a useful performance by taking all possible co-dependencies between three genes (including three-way dependencies) into account simultaneously and may thereby somewhat compensate the lack of post-transcriptional information.

Second, on real data, we identified another obstacle for motif detection. While positive correlations between interacting genes (indicating activation) are found frequently, strong negative correlations (indicating repression) are virtually absent. This lack of negative correlations has been ascribed to the fact that interactions involving repression show less consistency in measurements [30, 32]. It is thus especially difficult to detect motifs that involve repressing interactions. Examples for important motifs reported to involve repressing interactions are for instance the Lac system (*CRP*:TF, *Lacl*:TF, *lacZYA*:TG), Maltose utilization (*CRP*:TF, *mall*:TF, *malXY*:TG), and Methionine biosynthesis (*metJ*:TF, *metR*:TF, *metA*:TG) [33]. In addition, community wide assessments demnonstrated that network inference approaches are unable to infer the direction of regulatory relationships based on gene expression data alone [28]. The ANOVA based technique presented here is also not able to infer directions. However, in most regulatory relationships only one of the two partners is a TF such that regulation is obviously directed from the TF to the TG. We cannot resolve cases of interactions between two TFs, which correspondingly reduces our reported performance.

Besides evaluating the performance, we also examined motifs that were misclassified by 3WA. Such misclassifications might be linked to motifs showing atypical or unexpected behavior. We hypothesize that unexpected behavior might be due to one of the following three causes. First, not all gold-standard network motifs are correct due to incomplete or incorrect interactions in experimentally derived networks. A second reason for misclassifications might be that edges are weak, e.g. that the direct effect of the TF on the TG in a FFL motif is small. Here, an FFL motif might actually rather appear as a CSC motif. Finally, densely connected motifs might also exhibit a behavior different from isolated motifs if they are affected by strong inputs from the network [19, 34]. We therefore examined cases of misclassification in which strong deviations from the expected behavior of a motif are observed, i.e. misclassifications in which 3WA exhibited very high confidence values. In several such cases we presented literature evidence suggesting that the behavior of the motif as predicted by 3WA might be correct. We conclude that an analysis of seemingly misclassified motifs can provide important hints on motif behavior and even about potential gold standard errors.

We have presented an approach that successfully detected and discriminated different types of network motifs. While we demonstrated an increased performance in comparison to previous approaches, there are several aspects that generally limit the ability of methods to detect motifs. As we demonstrated the importance to treat subnetworks as a whole, a first obvious improvement is to consider larger subnetworks containing more than 3 genes and interactions. Second, the incorporation of additional details on the interactions could lead to improvements, for instance by considering the sign (repression/activation) and the strength of interactions. Third, the temporal dynamics of interactions could be analyzed, perhaps by a dedicated treatment of time-series data. Finally, focusing the analysis on expression data alone is another limitation. The activity of the respective proteins is difficult to assess without knowledge of their concentration, the activation status and the localization. Transcriptome data is just an approximation for protein activity. While many other datatypes useful for inference such as TF binding sites, open chromatin, DNase hypersensitive sites (DHS; [35]), and ChIP-Seq (Chromatin ImmunoPrecipitation; [36]) are now available, they have not yet been systematically exploited for motif inference. Once the use of these data types in inference has been established, they can be incorporated into our proposed scenario.

As artificial datasets do not sufficiently emulate the properties of real data, we find that great care is required if subnetwork detection and interpretation approaches are developed and optimized primarily on artificial data. Despite the involved uncertainties (e.g. incompleteness of current gold standards, measurement errors, etc.), it might still be the best strategy to focus algorithm development efforts on real data.

## Materials and methods

*Data sources*. In this study we used three datasets from the DREAM5 (D5) competition [28] and one additional dataset from the M3D database [37] (Table 1). DREAM5 was a blinded GRN inference challenge in which the participants inferred the underlying network using the microarray expression data. The data consists of expression profiles of genes measured in different experimental conditions such as drug, environmental and gene perturbations, or a combination of them. For experiments that were provided as time course measurements, we considered each time-point as a separate condition. Details regarding the datasets and the preprocessing procedure (e.g. computation of fold changes) can be found in Table 1 and Küffner et al. [7].

**Table 1:** DREAM5 (D5) and M3D datasets used in this study.

Dataset	# TF	# Genes	# TF pert.	# Genes pert.	# Chips	# Interactions
Artificial (D5)	195	1643	38	38	805	4012
*E*. *coli* (D5)	334	4511	20	43	805	2066
*E*. *coli* (M3D)	167	4297	17	67	907	2066
*S*. *cerevisiae* (D5)	112	6777	7	12	904	3742

The dataset contain a large number of gene expression measurements (chips) for thousands of genes (# Genes) and hundreds of TFs (# TFs). Measurements are also done for a number of gene-specific perturbations (# TFs pert. and # Genes pert.). The number of annotated gene regulatory interactions in the respective underlying networks is shown with # Interactions. All data was used as provided by the original sources and publications (see text).

*Gold standard of network motifs*. True motifs were extracted from available gold standards of TF:TG interactions. In case of *E*. *coli*, we used RegulonDB [38, 39]. The regulatory network of *S*. *cerevisiae* was automatically derived by genome-wide chromatin immunoprecipitation data post-processed by conservation-based motif discovery algorithms [40]. In case of DREAM5 artificial data, an artificial regulatory network was used by the challenge organizers to simulate the expression profiles. Usually, as not all regulatory mechanisms (like post-transcriptional events) and their precise parameters are implemented in simulators, the artificial data often cannot reflect the reality of the cell. On the other hand, the gold standard of real datasets is also not free from errors, i.e several true interactions are missing and false positives are introduced due to noise in experimental measurements.

### Detection of dependencies using ANOVA

*Analysis of Variance*. ANOVA is a technique to evaluate combinations of several independent variables or factors and to identify those that have a significant effect on the value of a response variable. Generally, the gene expression (response variable) varies in response to experimental conditions (C) as factor. A factor has different “groups” or “levels”, e.g. sets of replicated measurements representing knock-outs, over-expressions, or chemical treatments. The one way ANOVA (1WA) and t-test both test the null hypothesis that the population means across several groups of a single factor are equal (only two groups in case of the t-test). For instance, 1WA is often employed to test for the significance of differential gene expression across conditions, with *μ*_*rc*_ being the gene expression of *r*-th replicate, *r* ≤ |*R*|, of the *c*-th experimental condition, *c* ≤ |*C*|. |*R*| and |*C*| are the number of replicates and experimental conditions, respectively.

To test the null hypothesis (i.e. no differential expression), two variances, i.e. “between-group variance” and “within-group variance”, are computed. The “between-group variance” or “explained variance” is the variance of group means (${\bar{y}}_{.c}$) from total mean (${\bar{y}}_{‥}$). The “within-group variance” represents the variance between replicates of the same group, and is considered to quantify measurement error. Formulas for 2WA are given in [41].

In case of 1WA, these deviations can be written as:

$$SS_{T}=\sum_{r=1}^{\left|R\right|}\sum_{c=1}^{\left|C\right|}{\left(y_{rc}-{\bar{y}}_{‥}\right)}^{2}=\left|R\right|\sum_{c=1}^{\left|C\right|}{\left({\bar{y}}_{.c}-{\bar{y}}_{‥}\right)}^{2}+\sum_{r=1}^{\left|R\right|}\sum_{c=1}^{\left|C\right|}{\left(y_{rc}-{\bar{y}}_{.c}\right)}^{2},$$ (1)

where *SS*_*T*_ is defined as the sum of squared (SS) deviations of all replicates from overall mean. *SS*_*T*_ can thus be expressed as the sum of two sum of squares:

$$SS_{T}=SS_{C}+SS_{error},$$ (2)

**Fig 9:**
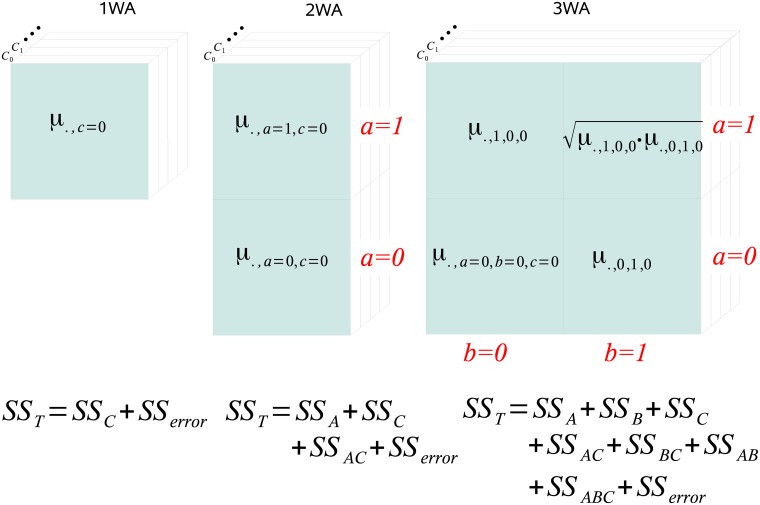
The design of *N*-way ANOVA (NWA) illustrated for *N* = 1,2 and 3. The design of N-way ANOVA (NWA) illustrated for *N* = 1,2 and 3. Generally, ANOVA models experimental observations *μ* as responses to *N* involved factors. For example, 3WA models the response to three factors *A*, *B*, and *C* in form of *μ*_*r*,*a*,*b*,*c*_, where *a*, *b*, and *c* are indices of the factors *A*, *B*, and *C*, respectively. The first index, i.e. *r*, refers to the experimental replicates. Here, *A* and *B* have exactly two levels (*a* = 1 or 0, and *b* = 1 or 0), and *C* has as many levels as experimental conditions (*c* = 0, 1,…). Correspondingly, for lower dimensions, i.e. 2WA and 1WA, respective indices are skipped, i.e. we have *μ*_*r*,*a*,*c*_ for 2WA and *μ*_*r*,*c*_ for 1WA. N-way ANOVA then has *N* + 1 dimensions, where the extra dimension represents the experimental replicates. Thus, each field (blue square) represents a set of replicate measurements. Then, a one-way ANOVA (1WA) tests if *μ* exhibits significant differences across conditions (index *c*), and has often been applied to test for differential gene expression. 2WA has been successfully applied to network inference as it furthermore tests for the dependence of a TF (index *a* = 1) and a TG (index *a* = 0). 3WA models *μ*_*r*,*a*,*b*,*c*_ and, thus, tests for the dependence and independence of three factors. These factors model the influences of two transcription factors *TF*_*X*_ (index *a*) and *TF*_*Y*_ (index *b*) in addition to the experimental conditions. Consequently, *μ*_.,0,0,0_, *μ*_.,1,0,0_ and *μ*_., 0,1,0_ represent the mean of the replicate measurements of *TG*_*Z*_, *TF*_*X*_ and *TF*_*Y*_ in condition *c* = 0, respectively. The common activity of *TF*_*X*_ and *TF*_*Y*_ is imputed as geometric mean. The total sum of squares (*SS*_*T*_) is composed of 2^*N*^ sums of squares (SS) which represent influences of individual and combined factors (bottom of Figure).

More generally, an N-way ANOVA tests the effect of *N* factors, as well as the joint effect of combinations of *k* ≤ *N* factors on the response variable. For example, 1WA decomposes the *SS*_*T*_ into two terms, namely the *SS*_*within*_ and *SS*_*error*_. Consequently, 1WA, 2WA, 3WA,…, NWA partition the *SS*_*T*_ into 2, 4, 8,…, 2^*N*^ SS terms, respectively (see Fig 9).

The variance is computed by dividing SS terms by the respective degree of freedom (*df*; the number of data points minus 1). An *F*-value is computed by weighting the explained variance against the error variance. *F*-values follow the *F*-statistics, which can be used to express the statistical significance of the involved factors as *p*-values. For instance, to estimate the significance of differential expression across conditions we compute *F*_*C*_ by:

$$F_{C}=\frac{Variance_{error}}{Variance_{within}}=\frac{SS_{error}/df_{error}}{SS_{within}/df_{within}},$$ (3)

To test a putative interaction between the gene pair *TF*_*X*_: *TG*_*Z*_, where *TF*_*X*_ and *TG*_*Z*_ are the transcription factor (TF) and the target gene (TG), respectively, we previously used 2WA [7] with two factors, *C* (conditions) and *A* (genes), i.e. the data is structured into a three dimensional matrix of genes (here always two, the TF and the TG), the experimental conditions, and the corresponding replicates. Here, *A* and *C* describe the effects on the expression measurements derived from the genes and conditions, respectively. Thus, *SS*_*A*_ and *SS*_*C*_ quantify the variance in the expression profiles across *C* and *A* respectively. $\eta_{C}^{2}$ computes the degree of dependency between *TF*_*X*_ and *TG*_*Z*_

$$\eta_{C}^{2}=\frac{SS_{C}}{SS_{T}},F_{\eta }=\frac{V_{C}}{V_{T}},$$ (4)

which is proportional to the fraction of the total variance that is explained by the differential expression across experimental conditions. $\eta_{C}^{2}$ is a measure of dependency that, in contrast to for example Pearson’s correlation, is non-parametric, and non-linear (see [7] for details). To infer the GRN, candidate TF:TG interactions are ranked in descending order of $\eta_{C}^{2}$.

*Analysis of three-gene motifs using 3way-ANOVA*. This paper focuses on the analysis of three-gene motifs with two TFs *TF*_*X*_ and *TF*_*Y*_ and the target gene *TG*_*Z*_. In a three gene structure, the number of possible interactions is increased from one in the pairwise scenario to three (*TF*_*X*_:*TF*_*Y*_, *TF*_*X*_:*TG*_*Z*_, *TF*_*Y*_:*TG*_*Z*_). We therefore propose to extend 2WA to 3way-ANOVA (3WA) to measure the strength of dependencies in a three-gene structure. The 3 dimensions of 3WA are *A*, *B*, and *C* which model the TFs *TF*_*X*_ and *TF*_*Y*_ and conditions, respectively. This allows us to extend the model from the effect of a single TF and the conditions *C* on the expression of *TG*_*Z*_ (2WA), to the individual as well as joint effects of two TFs and *C* on *TG*_*Z*_ (3WA). See Fig 9 for a detailed descriptions of the N-way ANOVA (NWA, for *N* = 1, 2 and 3) design, including the corresponding data matrices.

In 3WA, we compute 8 SS terms. Like in the applications of ANOVA mentioned above, 3WA aims to model the expression of target gene *TG*_*Z*_. We normalize the SS terms by dividing them by *SS*_*T*_, to obtain a ratio in the range [0‥1], and, thus, to make them comparable across motifs. In the following we briefly describe the intuition behind these terms, and their significance for motif analysis. *SS*_*A*_(*SS*_*B*_) measures the independence of the expression of *TF*_*X*_(*TF*_*Y*_) from the expression of remaining genes. As shown by Eq 4, SS terms do not only capture the variance, and, thus, the independence of expression, but can also express dependency. The sum of normalized terms is 1. Correspondingly, if *SS*_*AC*_ explains an increased part of the variance, less variance can be attributed to the relationship between *TF*_*Y*_ and *TG*_*Z*_. This in turn implies an increased dependency between *TF*_*Y*_ and *TG*_*Z*_. The same can be concluded for *SS*_*BC*_, i.e. it models the dependency between *TF*_*X*_ and *TG*_*Z*_. A high *SS*_*C*_ means that the total variance in the conditions is high, and, thus, the variance between the three genes is low, indicating that they are highly dependent. To exemplify, in case of 3way ANOVA, *SS*_*A*_ and *SS*_*AC*_ can be computed as follows:

$$SS_{A}=\left|R||B||C\right|\sum_{a\in A}{\left({\bar{y}}_{.a‥}-{\bar{y}}_{\ldots .}\right)}^{2},$$ (5)

$$SS_{AC}=\left|R||B\right|\sum_{a\in A}\sum_{c\in C}{\left({\bar{y}}_{.a.c}-\left({\bar{y}}_{\ldots .}+\left({\bar{y}}_{\ldots c}-{\bar{y}}_{\ldots .}\right)+\left({\bar{y}}_{.a‥}-{\bar{y}}_{\ldots .}\right)\right)\right)}^{2},$$ (6)

where, *y*_*rabc*_ is the response in replicate *r*, level *a* of factor *A*, level *b* of factor *B*, and level *c* of factor *C*. ${\bar{y}}_{\ldots .}$ is the overall mean, and ${\bar{y}}_{.a‥}$ is the mean of the response of factor *A* in level *a* across all other factors and replicates.

High *SS*_*AB*_ indicates that in a subset of conditions the expression pattern of *A* and *B* are different. Finally, *SS*_*error*_ and *SS*_*ABC*_ refer to unexplained variance, and, thus, represent error in the model. To the best of our knowledge, these last three terms have no positive evidence for motif detection.

**Fig 10:**
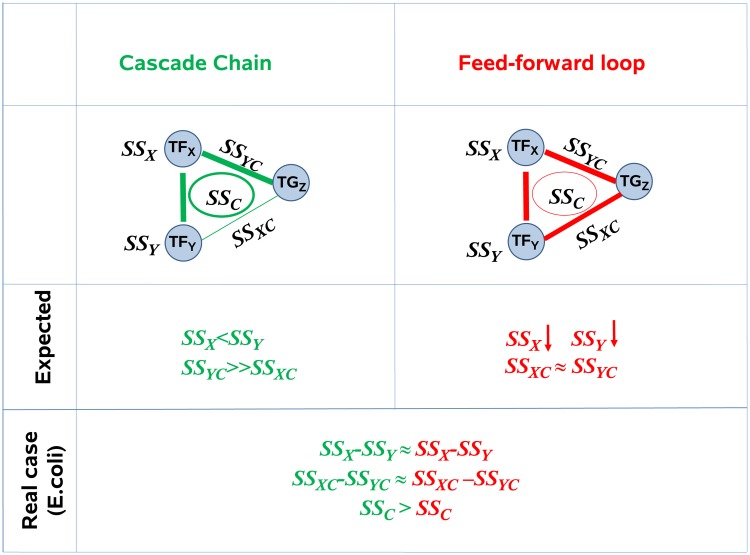
Sum of squares terms (SS) in a three way ANOVA and their meaning for network motif detection. FFL and CSC motifs are shown in red and green, respectively. *TF*_*X*_, and *TF*_*Y*_ are the TFs and *TG*_*Z*_ is the regulated target gene. Here, we show how SS terms can be used to distinguish FFL and CSC motifs and compare an artificial dataset and an *E*.*coli* dataset. In the artificial data, *SS*_*BC*_ is higher compared to *SS*_*AC*_ in CSC motifs, assuming that the interaction between *TF*_*Y*_ and *TG*_*Z*_ is absent in the CSC motif. In case of FFL, due to the edge between both *TF*_*X*_: *TG*_*Z*_ and *TF*_*Y*_: *TG*_*Z*_ pairs, we expect *SS*_*AC*_ ≈ *SS*_*BC*_. In CSC motifs, *SS*_*B*_ is higher than *SS*_*A*_, because *TF*_*Y*_ itself is regulated independently while *TF*_*X*_ depends on *TF*_*Y*_. See Methods for a description of the various SS terms and their meaning.

In Fig 10, we illustrate in a toy example (see for the toy model) the terms suitable to differentiate between the behavior (independencies and dependencies) of FFL and CSC motifs and are thus used to examine their behavior in the following. For sake of simplicity, in the toy dataset motifs are assumed to be independent of the whole network, while in reality, environmental signals as well as local signals from the network itself, e.g. the regulatory effect of other TFs, affect the expression of genes in the motif. See Results for a description on how these terms are used to distinguish motifs on actual data.

In summary, 3WA considers all the three genes and their putative (in)dependencies at the same time. 3WA can be considered in form of a function which takes three arguments of the type of expression profile of genes. For example, in function 3WA(*X*,*Y*,*Z*), *X*, *Y*, and *Z* are the expression profiles of the corresponding genes, typically *X* and *Y* being the transcription factors and *Z* being the target gene. See section 4.1.2 for the exact detail on how this function is used in a dataset-specific manner. In the context of distinguishing motifs with 3 genes, this feature of 3WA is in contrast to pairwise dependency coefficients (see Section 4.2) that analyze one interaction at a time and thus neglect the potential combinatorial influence of two TFs on the regulation of the expression of the TG.

#### Toy model

**Fig 11:**
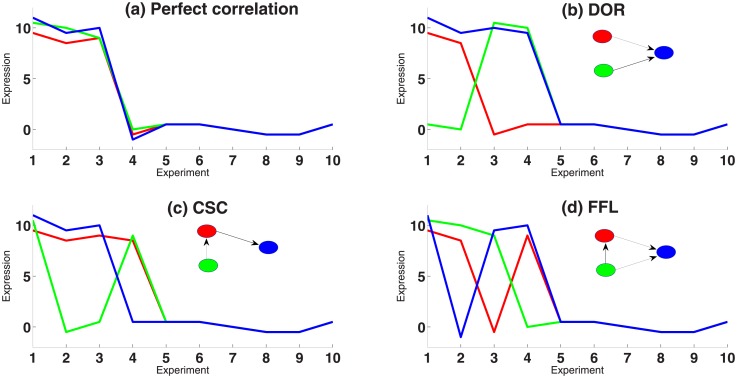
Four toy datasets for three genes each to simulate the expression pattern of network motifs. Four toy datasets for three genes each to simulate the expression pattern of network motifs. (a) A dataset where all three genes are highly correlated. (b-d) Three datasets which are designed to exhibit characteristics of the three motifs of CSC, DOR and FFL, respectively.

We created four toy datasets with three genes each. The first dataset represents a case where all three genes are highly correlated. The remaining three datasets were designed to exhibit characteristics of the three motifs of CSC, DOR and FFL, respectively. We used the toy data as shown in Fig 11 to examine the sum of squares (SS) terms resulting from a 3Way-ANOVA. Our observations and their comparison with *E*. *coli* dataset are shown in Fig 10. For instance, while in a DOR both TFs need to be active in order to turn on a TG, the TG in a CSC motif is more dependent on its immediate TF (red) than on the indirect second TF. Thus, we artificially designed the datasets to represent properties we expected to emerge from the motifs rather than properties of real datasets.

#### Modeling of motifs via combinations of sum of square terms

According to Fig 10, the toy dataset shows different behavior compared to *E*. *coli* dataset (see Section 2.1 for a comparison between characteristics of real and artificial datasets). Hence, we suggest to use different set of SS terms to classify motifs across datasets, and consequently, the combination of SS terms to classify motifs via 3way ANOVA should be carefully designed for new datasets. In particular, as artificial data, *E*. *coli* data and *S*. *cerevisiae* data are different (see Results), different combinations of SS terms need to be exploited to optimally detect motifs. Here we used three-fold cross-validation to determine the set of terms in order to avoid over-fitting. The set of terms used for pairwise classification of motifs across datasets are shown in Table 2.

**Table 2:** The set of terms used for pairwise classification of motifs across datasets.

Dataset	DOR vs. CSC	CSC vs. FFL	FFL vs. DOR
*in silico* (D5)	*SS*_*ac*_, 3WA(*TF*_*X*_,*TF*_*Y*_,*TG*_*Z*_)	*SS*_*bc*_, 3WA(*TF*_*X*_,*TF*_*Y*_,*TG*_*Z*_) using weights (see [7])	*SS*_*c*_/(*SS*_*ac*_ * *SS*_*bc*_), 3WA(*TF*_*X*_,*TF*_*Y*_,*TG*_*Z*_)
*E*. *coli* (D5)	1/*SS*_*ac*_, 3WA(*TF*_*Y*_,*TG*_*Z*_,*TF*_*X*_)	*SS*_*c*_ * *SS*_*bc*_/*SS*_*ac*_, 3WA(*TF*_*X*_,*TG*_*Z*_,*TF*_*Y*_)	*SS*_*ac*_/*SS*_*bc*_, 3WA(*TF*_*Y*_,*TG*_*Z*_,*TF*_*X*_)
*E*. *coli* (M3D)	1/*SS*_*ac*_, 3WA(*TF*_*Y*_,*TG*_*Z*_,*TF*_*X*_)	*SS*_*c*_ * *SS*_*bc*_/*SS*_*ac*_, 3WA(*TF*_*X*_,*TG*_*Z*_,*TF*_*Y*_)	*SS*_*ac*_/*SS*_*bc*_, 3WA(*TF*_*Y*_,*TG*_*Z*_,*TF*_*X*_)
*S*. *cerevisiae* (D5)	*SS*_*a*_ * *SS*_*ac*_/*SS*_*ab*_, 3WA(*TF*_*X*_,*TF*_*Y*_,*TG*_*Z*_) using weights (see [7])	*SS*_*b*_/*SS*_*ab*_, 3WA(*TF*_*X*_,*TG*_*Z*_,*TF*_*Y*_) using weights (see [7])	*SS*_*ab*_ * *SS*_*bc*_/(*SS*_*b*_ * *SS*_*ac*_), 3WA(*TF*_*X*_,*TG*_*Z*_,*TF*_*Y*_)

### Basic approaches

*Pairwise dependency coefficients*. Pairwise dependency coefficients have been widely used to infer GRNs. For the inference of GRNs, gene pairs are ranked based on the dependency between their expression profiles, so that interactions with higher dependency are considered to be more confident. One of these dependency measures is 2WA, which we used to infer artificial and real networks of the DREAM5 challenge [7]. Pearson’s correlation (PC), Spearman’s correlation (SP), and mutual information (MI) are other examples of pairwise dependency measures [5, 6]. Among these measures, PC is limited to measuring linear dependencies. SP is similar to PC, but uses the ranks instead of the values. MI measures the amount of information shared among a number of variables. The MI between variables *X* and *Y* is computed as follows:

I(X,Y)=H(X,Y)-H(Y|X)-H(X|Y), (7)

where *H* denotes the entropy, and *H*(*X*, *Y*) is the joint entropy between *X* and *Y* and measures the actual amount of information in *X* and *Y*. *H*(*X*|*Y*) is the amount of information in *X*, if *Y* is known, i.e. the amount of information in *X* which is independent of *Y*. Hence, *I*(*X*, *Y*) represents the amount of redundant (shared) information between *X* and *Y*.

**Fig 12:**
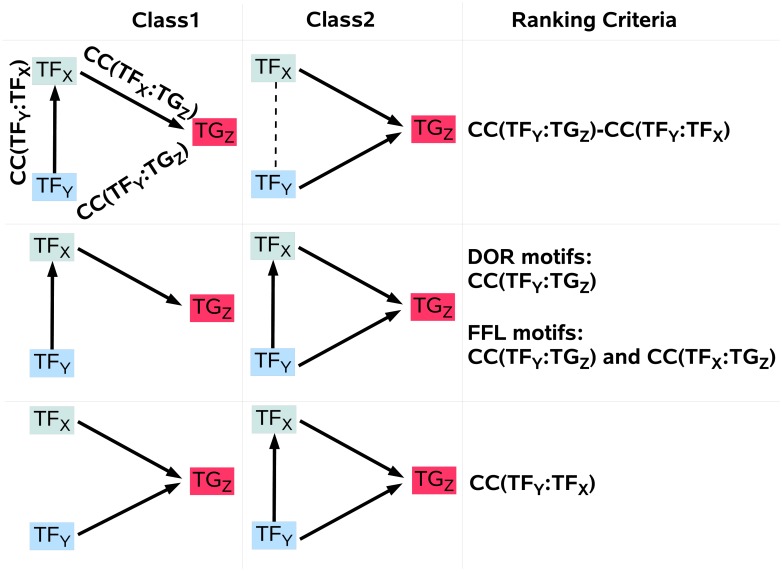
Using pairwise dependency coefficients for pairwise distinguishing of motifs. Using pairwise dependency coefficients (CC) for pairwise distinguishing of motifs. *CC*(*TF*_*X*_, *TF*_*Y*_) shows the pairwise dependency between *TF*_*X*_ and *G*_*Y*_. The criteria to distinguish pairs of motifs using CC is shown for each case separately.

To distinguish three-gene motifs based on pairwise dependency coefficients, we rank motifs by comparing the pairwise dependency of their discriminating edge(s) (Fig 12). For example, to distinguish CSC from FFL, motifs are ranked according to the pairwise dependency of interacting TF:TG pairs in FFL, and the non-interacting TF:TG pair in CSC. Assuming relative independence of both motifs from other regulating signals, we expect that both TF:TG edges in FFL show stronger dependency compared to the non-interacting TF:TG pair in CSC. However, in a CSC motif the pair *TF*_*Y*_: *TG*_*Z*_ might be highly correlated due to the indirect interaction through *TF*_*X*_, making it hard to be distinguished from a FFL motif (Fig 12b).

*Conditional dependency coefficients*. Conditional dependency coefficients measure the dependency between two variables (here, between the expression profiles of a TF:TG pair) conditioning on one or more other variables (other TFs). Conditioning means to reduce the dependency by the amount that is due to the other variables. For example, in a CSC motif (Fig 1) conditional dependency coefficients can be used to measure the correlation of the pair *TF*_*X*_:*TF*_*Y*_, after conditioning over *G*_*Z*_. Pearson’s correlation or Spearman’s correlation of *TF*_*X*_:*TF*_*Y*_ pair conditioned on *TG*_*Z*_ are computed as follows

$$r_{G_{X}G_{Y}|G_{Z}}=\frac{r_{TF_{X}TF_{Y}}-r_{TG_{Z}TF_{Y}}r_{TG_{Z}TF_{X}}}{\sqrt{\left(1-r_{TG_{Z}TF_{Y}}^{2}\right)\left(1-r_{TG_{Z}TF_{X}}^{2}\right)}},$$ (8)

where *r*_‥_ is the pairwise correlation, and $r_{G_{X}G_{Y}|G_{Z}}$ is the correlation between parts of *TF*_*X*_ and *TF*_*Y*_ that are uncorrelated with *TG*_*Z*_. High $r_{TF_{X}TF_{Y}|TG_{Z}}$ values can be an indication of a direct interaction rather than an indirect.

Conditional mutual information is not only dependent on the pairwise dependency between the three variables, but also on the independence of *X*, and the overall dependency between the three variables.

H(Y,X|Z)=H(X,Z)+H(Y,Z)-H(Z)-H(X,Y,Z), (9)

where *H*(*X*, *Z*), *H*(*Y*, *Z*), *H*(*X*, *Y*, *Z*) are joint entropies, and *H*(*X*, *Y*|*Z*) is the amount of information in *Y* and *X* after variable *Z* is known.

### Evaluation of results

We considered the problem of motif inference as three classification problems, in each we distinguished a pair of motifs (two-class problem). To evaluate the performance of each classification, we ranked the predictions by decreasing score and computed the area under the ROC curve (AUROC) and the area under the precision recall curve (AUPR). The AUROC estimates the probability that a predictor will rank a randomly chosen positive instance higher than a randomly chosen negative one. Sensitivity, also known as recall, estimates the probability that the label of a positive sample is correctly identified. Precision measures the percentage of true positives (TPs) that are correctly predicted. Since AUROC = 50% corresponds to random guessing, only values between 50% and 100% are shown.

### Applicability of ANOVA

ANOVA, typically applied to detect statistically significant differences between means of groups of data values, rests on two basic assumptions. The first assumption is that the values are normally distributed and second, that the groups have the same variance. Indeed, as we showed previously [30], the distributions of the four underlying sets of data used in this work are appoximately normal (Fig A in S1 File) and that then ANOVA based techniques are appropriate. We thus consider the first assumption to hold.

The second assumption is related to the fact that the F statistic used by ANOVA to assess significance may under/overestimate significance levels when smaller/larger groups (respectively) exhibit larger variance within the dataset. In such cases, the F-statistics might result in biased probability estimates. We performed Levenes test in order to test for the second assumption of homogeneity of variance. Here, we use the same partitioning of samples into groups that was used in 3WA itself (compare Fig 9). Indeed we find that the variance is not homogeneous between samples and that probability estimates derived from the the F-statistics may be biased.

However, we argue that deviations from the assumption of homogeneity of variance will have only limited influence on the results of this study. First, the number of samples that we use in the ANOVA is very high (between 800-900 samples) such that even small differences in variance may result in significant Levene’s test p-values. We find that the actual differences in variance are rather small. Fig B in S1 File shows the data distribution for the motifs with most and least significant Levenes test p-value.

Furthermore, our method aims to assess and prioritize motifs based on their relative rank, based on ratios of sum of squares (referred to as eta-squared). It does not rely on the levels of significance of differences between the group means.

Finally, we employ the same paritioning of samples into groups for each of the tested motifs such that biases from heterogeneous variance likely affect each motif in a similar way and will have little influence on the overall ranking of motifs.

## Supporting information

S1 File ANOVA Results- Supplementary results assessing the applicability of ANOVA. (PDF)
