# Supplementary material for: Detection of network motifs using three-way ANOVA
Source: PLoS One. 2018 Aug 6;13(8):e0201382. doi: 10.1371/journal.pone.0201382 (PMC6078297; doi:10.1371/journal.pone.0201382)
Supplement: S1 File — (PDF) [file pone.0201382.s001.pdf]

1 Applicability of ANOVA

ANOVA rests on two basic assumptions. The first assumption is that the values are normally distributed and second, that the groups have the same variance. Indeed, the distributions of the underlying sets of data used in this work are all approximately normal (Figure A). We thus consider the first assumption to hold.

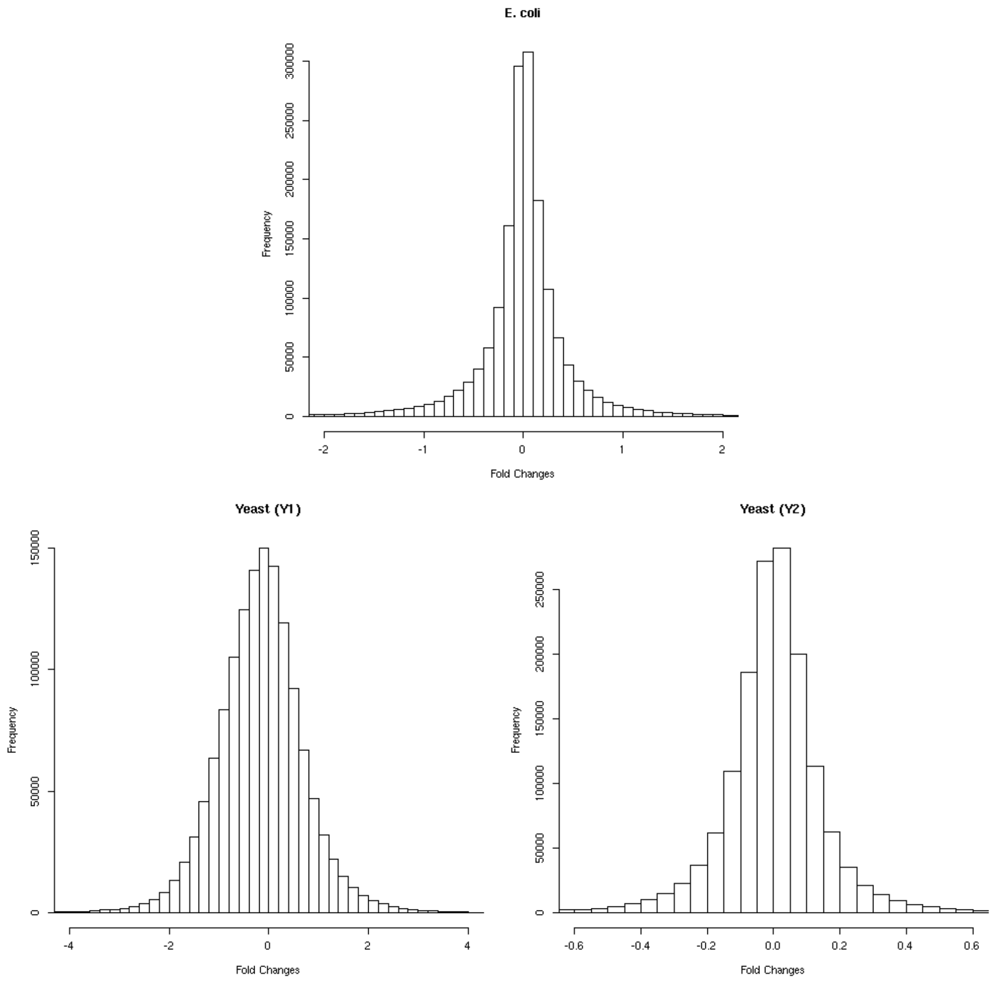

Figure A: Distribution of log fold changes (x-axis). The y-axis displays the number of fold changes observed in a particular bin. The histograms represent the distributions for the expression compendia used in our study.

The second assumption is related to the fact that the F statistic used by ANOVA to assess significance may under/overestimate significance levels when smaller/larger groups (respectively) exhibit larger variance within the dataset. In such cases, the F-statistics might result in biased probability estimates. We performed Levene's test in order to test for the second assumption of homogeneity of variance. Here, we use the same partitioning of samples into groups that was used in 3WA itself (compare Figure 9). Of the 1003 motifs tested, roughly half (519) exhibit a Bonferroni-corrected p-value of 0.01 or less, and thus exhibit variance that is not homogeneous between samples. However, we find that the actual differences in variance are indeed rather small, as depicted in Figure B that shows the data distribution for the motifs with most and least significant Levene's test p-value.

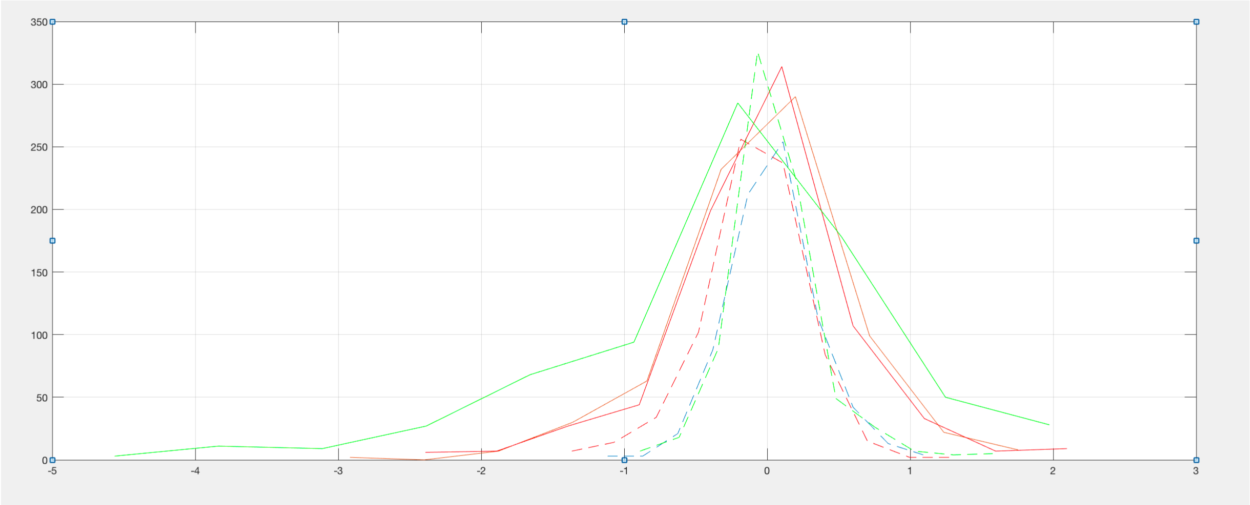

Figure B: Distribution of log fold changes (x-axis) for the motif with highest (p=1.0, shown by solid lines) and lowest ($p=2.22*10^{-27}$, shown by dashed lines) p-values, respectively. The y-axis displays the number of fold changes observed in a particular bin.
